# Research on predictive model for tracheal tube sizes in adult double-lumen endotracheal intubation based on radiomics and artificial intelligence

**DOI:** 10.3389/fmed.2025.1657138

**Published:** 2025-10-06

**Authors:** Shaopeng Ming, Zhaoyu Li, Shu Yan, Wei Lan, Hongtao Liu, Yanzhuo Zhang

**Affiliations:** ^1^Department of Anesthesiology, Liuzhou Workers Hospital/The Fourth Affiliated Hospital of Guangxi Medical University, Key Laboratory of Anesthesia and Perioperative Neuroprotection of Liuzhou, Perioperative neuroprotection Engineering Technology Research Center of Liuzhou, Liuzhou, China; ^2^School of Public Health and Management, Guangxi Health Science College, Nanning, China

**Keywords:** radiomics, artificial intelligence, double-lumen endotracheal intubation, tracheal tube size prediction, CT imaging

## Abstract

**Objective:**

This study aims to develop a predictive model for tracheal tube sizes in adult double-lumen endotracheal intubation using radiomics and artificial intelligence (AI) technologies to enhance the safety and efficiency of intubation procedures.

**Methods:**

A retrospective study design was adopted. Computed tomography (CT) imaging data of the neck and chest from 500 adult patients were collected, and radiomic features were extracted. After a rigorous screening, 390 patients were included in the analysis. Radiomics techniques were applied to analyze CT images and extract features related to tracheal tube size selection. Predictive models were constructed using AI algorithms, including random forests, decision tree, support vector machines, and Baidu Wenxin ERNIE.

**Major results:**

Among the models constructed, the Baidu Wenxin ERNIE model exhibited the best predictive performance, achieving an accuracy of 0.77 on the test set. Primary evaluation metrics, including accuracy, precision, recall, and F1-score, were compared to determine the optimal predictive model.

**Conclusions:**

This study successfully developed a predictive model for tracheal tube sizes in adult double-lumen endotracheal intubation based on radiomics and AI, demonstrating high predictive accuracy. This model has the potential to provide clinicians with a convenient, rapid, and efficient method of airway assessment, thereby enhancing the safety and efficiency of double-lumen endotracheal intubation.

## Introduction

Double-lumen endotracheal tubes are a pivotal instrument in thoracic surgery, where their dual-lumen configuration effectively partitions diseased and healthy lung segments to enable single-lung ventilation, thereby accommodating diverse surgical requirements (1). However, the precise selection of appropriately dimensioned tubes has emerged as a determinant of procedural success. Excessively large tubes increase the risk of airway trauma; bronchospasm; and postoperative complications such as laryngeal edema (2), pneumothorax, or tracheal rupture (3). On the other hand, undersized tubes may compromise airway sealing, precipitating ventilation leakage, contralateral lung contamination, hypoxemia, or surgical discontinuation. Consequently, anesthesiologists face a formidable challenge in optimizing double-lumen tube (DLT) sizing, necessitating the synthesis of clinical acumen with patient-specific anatomical, physiological, and procedural variables to harmonize surgical efficacy with patient safety (4).

Traditional selection methods that rely solely on physician experience or simple anatomical parameters (e.g., height and bronchial diameter measurements) have significant limitations, particularly when encountering anatomical variations, emergency surgeries, or unusual patient positioning, where the risk of misjudgment increases sharply. Although recent studies suggest that ultrasonic airway measurements can assist in DLT selection with improved accuracy (5, 6), computed tomography (CT) offers distinct advantages such as minimal measurement error, superior depiction of air-tissue interfaces, and avoidance of airway distortion artifacts (7). Consequently, CT remains the gold standard method for airway anatomical assessment because of its unparalleled precision and reliability in delineating critical respiratory structures (8). However, anesthesiologists may have limitations in interpreting CT images, particularly when identifying complex anatomical structures. When determining the size of double-lumen endotracheal tubes, it is necessary to process multiparameter data such as airway diameter, bronchial angle, and other relevant measurements. This process is cumbersome and time-consuming and requires high data processing abilities and spatial imagination from physicians. To ensure accurate selection, doctors often need to repeatedly measure and compare data and comprehensively consider their experience, which inadvertently increases the time and effort required for preoperative preparation. Therefore, there is an urgent need for a more convenient, rapid, and accurate assessment method.

Radiomics is an interdisciplinary research direction in medicine and computer science that refers to the extraction of a large amount of imaging information from images (such as CT, MRI, and PET) and assisting physicians in making the most accurate diagnosis by means of deeper mining, prediction, and analysis of massive imaging data information (9). With the powerful algorithmic advantages of artificial intelligence, it is possible to achieve in-depth mining of massive radiomics data, thereby constructing effective predictive models.

**Figure 1:**
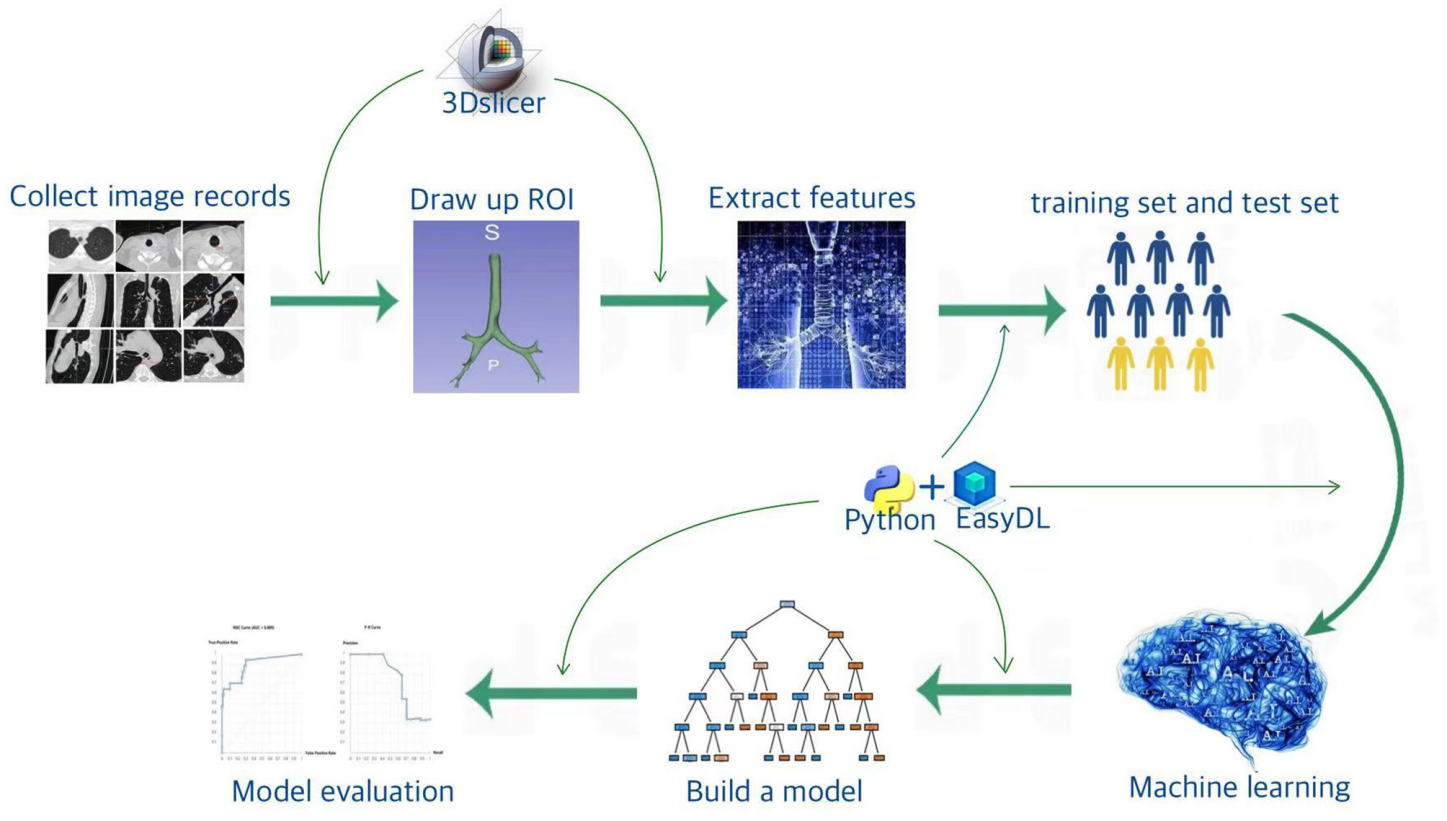
Flowchart of the research process.

The purpose of this study was to extract the radiomic features of the trachea from adult CT imaging data and establish a predictive model using artificial intelligence (machine learning) algorithms to leverage the powerful algorithmic advantages of artificial intelligence, thereby achieving intelligent prediction of double-lumen endotracheal tube sizes. This approach is simpler and faster than traditional manual measurements and achieves higher accuracy. A detailed flowchart of the research is shown in Figure 1.

## Methods

### Data acquisition

In the data acquisition phase, we meticulously collected CT plain scan images (in DICOM format) of the neck and chest from 500 patients aged 18 years or above at the Second Affiliated Hospital of Guangxi Medical University. Simultaneously, we comprehensively obtained the basic information of these patients, including multiple key indicators, such as age, sex, height, and weight.

Upon in-depth analysis of the 500 collected imaging data, we observed a pronounced imbalance in the judged catheter sizes. The number of patients judged to have a 28F catheter size was relatively large, while the number of patients judged to have a 41F catheter size was comparatively small. In machine learning modeling, such an imbalanced dataset poses significant challenges. Specifically, when modeling is based on this imbalanced dataset, the predictive results tend to be biased toward the majority class with a larger sample size. Consequently, samples from the minority class with a smaller sample size are more prone to misclassification than those from the majority class, which severely affects the model's predictive performance and accuracy (10).

Given that this study aimed to accurately predict most double-lumen catheter sizes, the excessive number of patients with a 32F catheter size in the collected data further exacerbated the data imbalance, adversely affecting the accuracy of the model. To effectively address this issue, we employed a random sampling method. From the final dataset, we selected 65 patients for each judged double-lumen catheter size. After rigorous screening, 390 patients were included in this study. Statistical analysis of the patient baseline characteristic data was performed using the Pandas library in Python.

### Ethics

Ethical approval for this study [Protocol No. 2024 - KY (1063)] was provided by the Medical Ethics Committee of the Second Affiliated Hospital of Guangxi Medical University, Guangxi, China (Chairperson: Prof. Xuyong Sun) on December 13, 2024.

### Study dates

The study was conducted from September 2024 to June 2025.

### Determination method for double-lumen endobronchial tube sizes

The anatomical characteristics of the right main bronchus make it prone to obstruction of the right upper lobe ventilation during intubation, while the structure of the left main bronchus facilitates the positioning and ventilation of the left-sided DLT. Thoracic surgeries often require left-sided one-lung ventilation, with the left-sided DLT being easier to position and having fewer complications, along with its extensive clinical application and a solid research foundation. Therefore, this study focused on the left-sided DLT as the research object.

**Figure 2:**
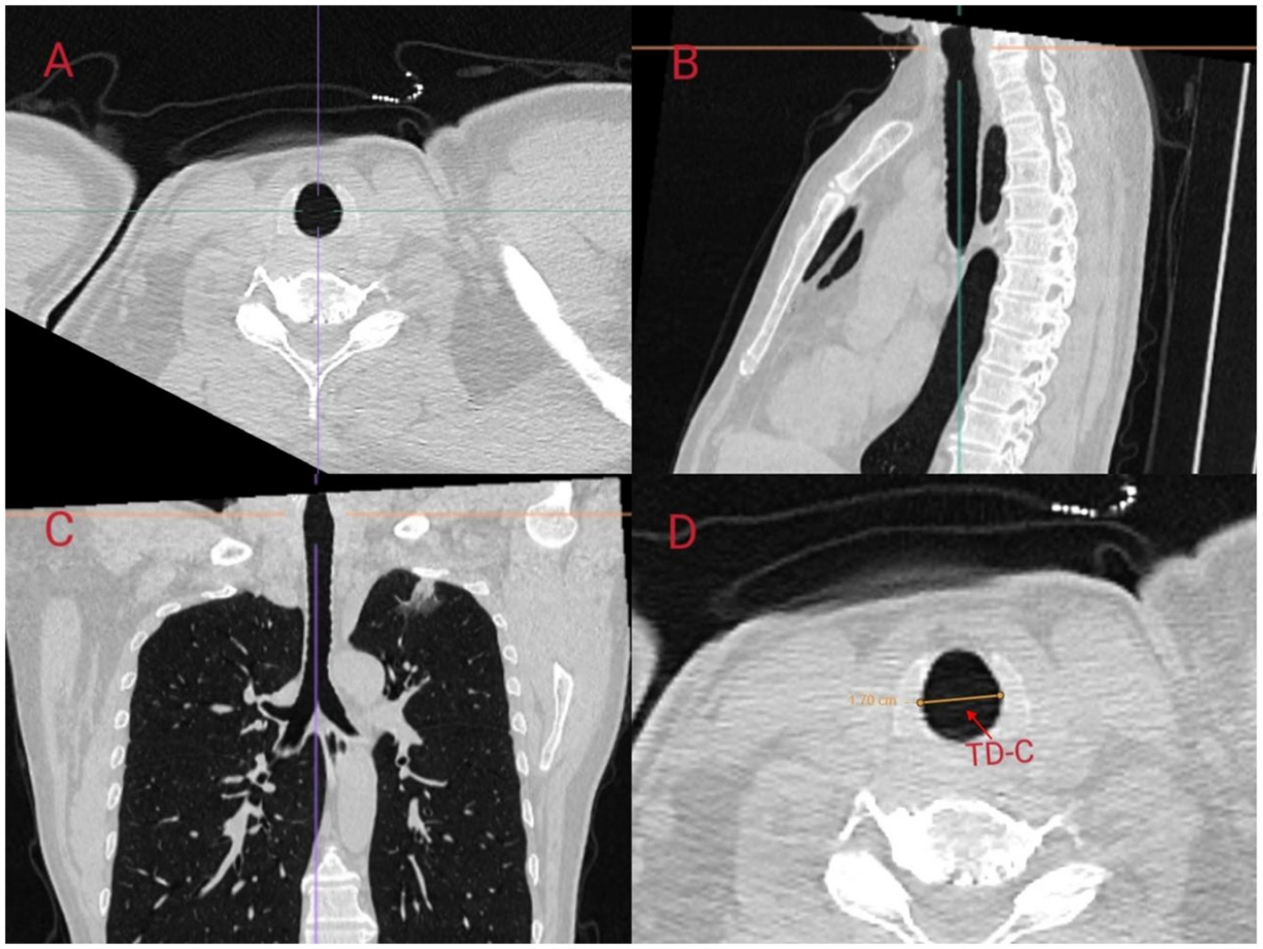
Scanning the left bronchus using multi-planar reconstruction (MPR) in Carestream PACS software. MPR of the cricoid cartilage was performed using **(A)** axial, **(B)** sagittal, and **(C)** coronal slices. The oblique angle of the transverse diameter of the cricoid cartilage was corrected in three dimensions to obtain strictly orthogonal cuts along the axis of the cricoid cartilage. The transverse diameter of the cricoid cartilage (TD-C) was measured using electronic calipers on the MPR image at the lower border of the cricoid ring **(D)**.

**Figure 3:**
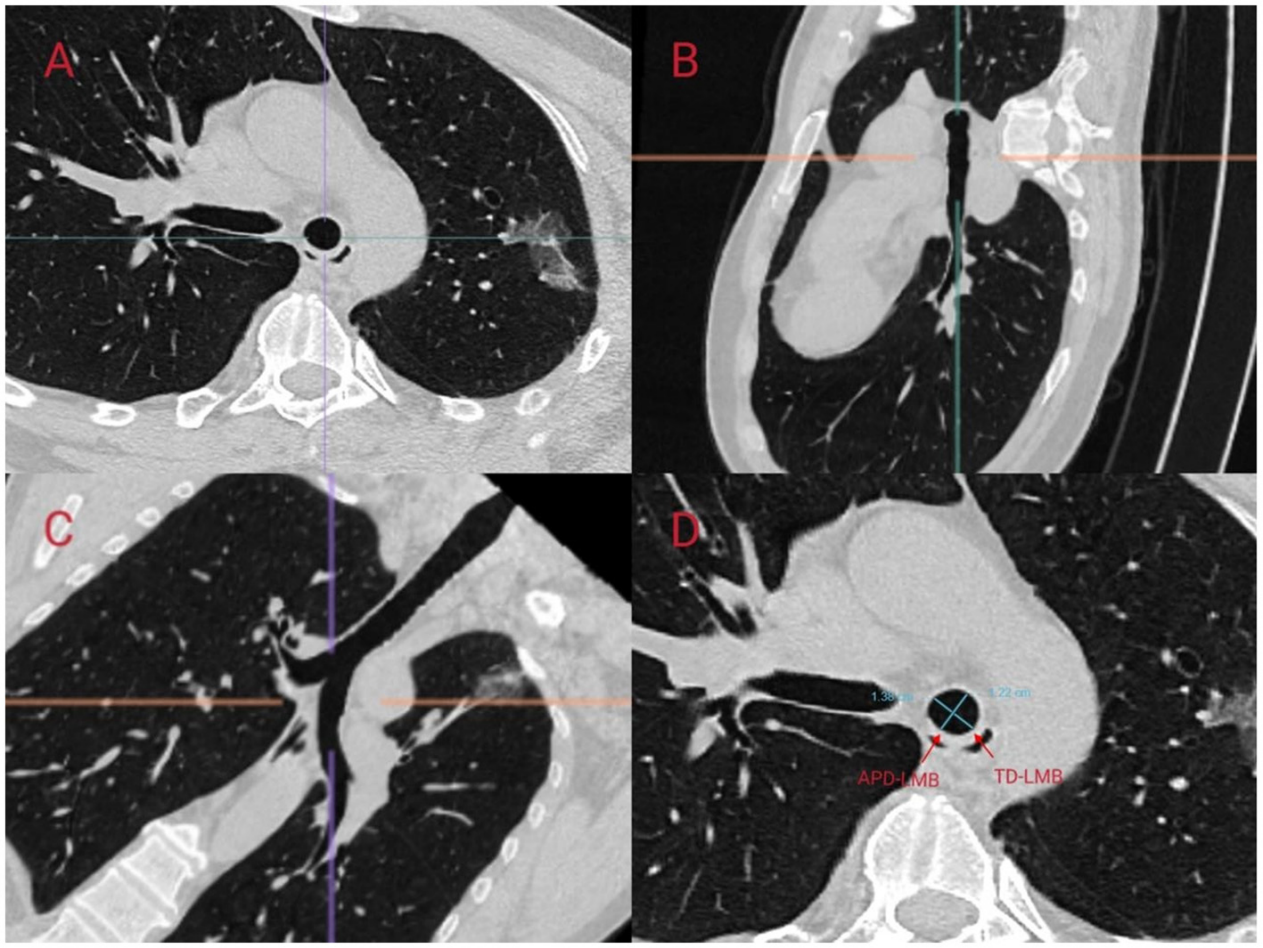
Scanning the left main bronchus (LMB) using Multi-Planar Reconstruction (MPR) in CarestreamPACS software to measure its diameter. MPR was performed using **(A)** axial, **(B)** sagittal, and **(C)** coronal slices. On the MPR image at 1 cm below the carina, the anteroposterior internal diameter of the left main bronchus (APD-LMB) and the transverse internal diameter of the left main bronchus (TD-LMB) were measured **(D)**. The equivalent circular diameter (ED-C) was calculated using the formula for the perimeter of an ellipse, π[3(a + b) – ([a + 3b] [3a + b])$\frac{1}{2}$], where a is the larger radius and b is the smaller radius, and the formula for the circumference of a circle.

We referred to the ISO 16628:2022 standard for specific parameters of the double-lumen endotracheal tubes. The use of right-sided DLTs may lead to an increased incidence of complications, such as intraoperative hypoxemia and postoperative atelectasis, due to the potential obstruction of the orifice of the right upper lobe bronchus. In contrast, left-sided DLTs are generally considered to have a higher safety profile and are thus the preferred choice for thoracic anesthesia (11). Therefore, in this study, we aimed to predict the appropriate size for left-sided DLTs. In a study conducted by Mathew et al., the accuracy of predicting the size of left-sided DLTs by measuring the transverse diameter of the cricoid cartilage using CT was 97.5%, which was significantly superior to the 75% accuracy achieved using traditional methods based on height and gender (12). Other studies have indicated that the diameter of the left bronchus is also of significant importance in predicting the appropriate size of left-sided DLTs (13). In this study, we predicted the size of left-sided DLTs in patients by measuring the transverse diameter at the level of the cricoid cartilage (TD-C) on CT scans and calculating the equivalent circular diameter (ED-C) of the left bronchus. We followed the measurement methodology outlined by Shiqing et al. (14) in which anesthesiologists trained by radiologists utilized the MPR module of the Carestream PACS software to perform multiplanar reconstruction and measurements of the patients' trachea using axial, sagittal, and coronal slices. The inclination of the cricoid cartilage and left bronchus was adjusted to obtain strictly orthogonal slices (Figures 2, 3).

Airway measurements corresponding to the selection of endotracheal tube sizes from other studies (15, 16) are presented in Table 1.

**Table 1:** Selection criteria for left double-lumen tubes (LDLTs) based on transverse diameter of the cricoid cartilage (TD-C) and equivalent circular diameter (ED-C) of the left bronchus.

**LDLT size**	**TD-C (mm)**	**ED-C (mm)**
28F	<12.5	<9
32F	≥12.5	≥9
35F	≥14	≥10
37F	≥15	≥10.5
39F	≥16	≥11.5
41F	≥18	≥12.5

The final selection is the smallest size that accommodates both criteria.

### Delineating the region of interest (ROI)

The region of interest (ROI), a designated image area marked within an image, served as the primary focus of subsequent analyses. Further in-depth analytical processing could be conducted by fixing this region. In this study, the ROI was localized within the mid-airway structures of the tracheobronchial tree. Specifically, it encompassed the entire segment of the tracheal lumen from the inferior border of the cricoid cartilage to the carina, as well as the proximal portions of both the main bronchi.

We employed a semi-automatic approach using 3DSlicer 4.11 (17) to delineate the ROI. The detailed steps are as follows.

**Figure 4:**
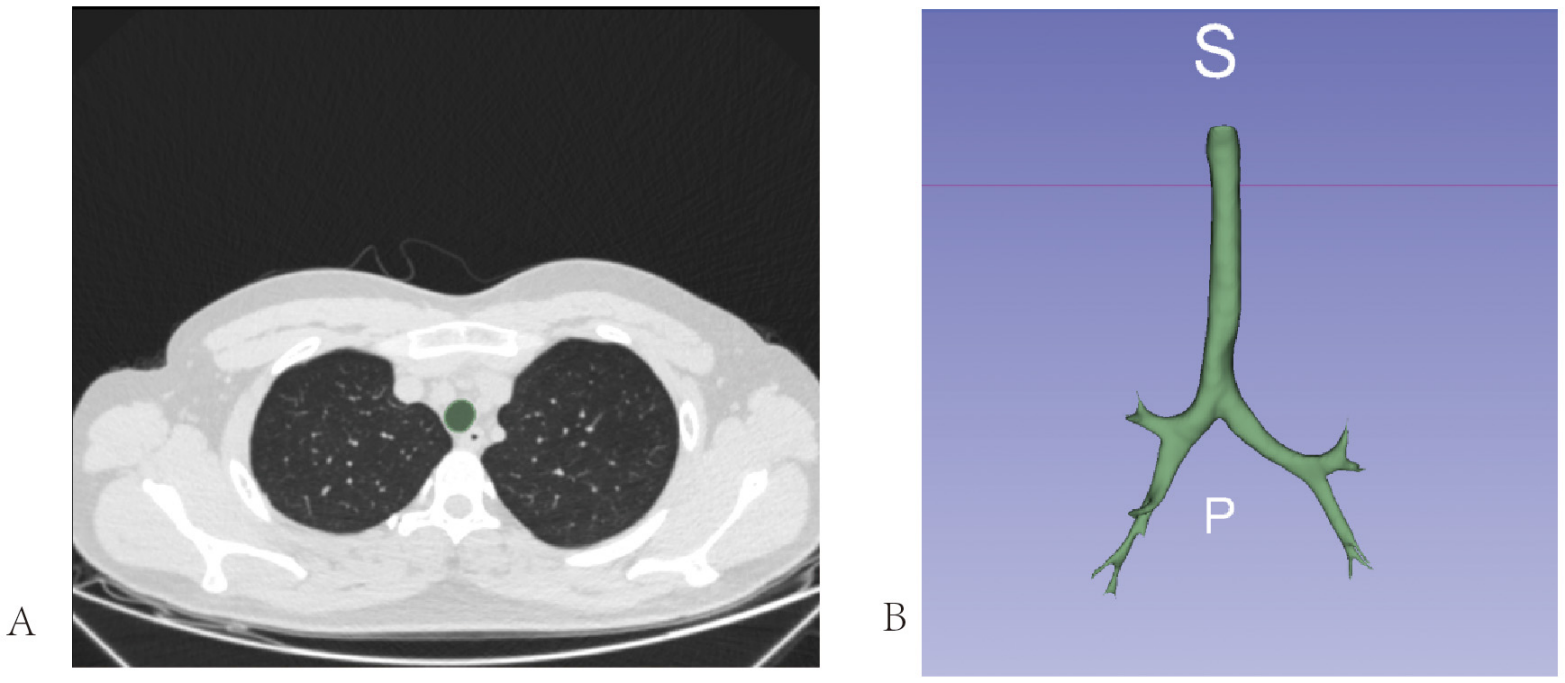
Extraction of the Region of Interest (ROI). **(A)** We utilize a horizontal tracing tool to delineate a single axial plane of the trachea. **(B)** The complete extracted ROI.

(1) We initiated the drawing process at the subglottic cricoid cartilage level using a level-tracing tool. This tool allows the generation of a contour within a plane by moving the mouse; all pixels on this contour share the same grayscale value as the pixel at the location of the mouse. Five consecutive planes were drawn in this manner (Figure 4A).
(2) Subsequently, we used the Fast Marching module within the SegmentEditorExtraEffects plugin to fill the drawn planes. We set the maximum parameter to 1% and selected a segment volume within a range of 3%−10%.

Following these steps, a three-dimensional ROI of the subglottic tracheal and bronchial airways was constructed (Figure 4B).

### Radiomics feature extraction

Based on the manual approach for tracheal tube selection, which indicates that the choice of tracheal tube is closely related to the tracheal shape rather than other radiomic features, we exclusively extracted radiomic features associated with shape, namely, shape3D (representing the 3D shape of the ROI and shape2D (denoting the 2D shape of the ROI). For other parameter settings, we adopted the software's default values: [Binwidth = 25 (for calculating and returning grayscale values) and Rsampledvoxel size = 1, 1, 1 (for specifying the output image spacing)].

As a result, we obtained the following 14 feature datasets: SurfaceVolumeRatio (the ratio of surface area to volume), Maximum2DDiameterColumn (the maximum 2D diameter in the column direction), Sphericity (a measure of how closely the shape resembles a sphere), MinorAxisLength (the length of the minor axis), SurfaceArea (the total surface area), Maximum2DDiameterSlice (the maximum 2D diameter in the slice direction), VoxelVolume (the volume of each voxel), Maximum2DDiameterRow (the maximum 2D diameter in the row direction), Elongation (a measure of how elongated the shape is), Flatness (a measure of how flat the shape is), Maximum3DDiameter (the maximum 3D diameter), MajorAxisLength (the length of the major axis), LeastAxisLength (the length of the least axis), and MeshVolume (the volume calculated from the mesh representation).

We consolidated all extracted features into a matrix, in which each row corresponded to a patient case and each column represented a radiomic feature. Given that Python requires the outcome variable to be of integer type, we converted the previously determined tracheal tube sizes (28F, 32F, 35F, 37F, 39F, 41F) into integer form, assigning the numbers 1, 2, 3, 4, 5, and 6, respectively. Subsequently, we incorporated the tracheal tube size into the last column of the matrix. We utilized the SlicerRadiomics (18) plugin within 3DSlicer 4.11 to extract the radiomic features.

### Quality control

Fifty CT images were randomly selected from the 390 CT images acquired in this study. Two senior radiologists (Radiologists A and B) independently segmented the airway lumen from the glottis to the carina and extracted shape-related radiomic features.

First, Radiologist A performed airway segmentation twice consecutively with a 1-week interval between the two sessions. Radiomic features were then extracted from each of the two segmentations, and the consistency of the extracted features was evaluated. Subsequently, Radiologist B independently segmented the airway and extracted the radiomic features. A consistency analysis was conducted between the radiomic features extracted by Radiologist B and those extracted by Radiologist A in the first session.

The intraclass correlation coefficient (ICC) was used for the consistency analysis. The following criteria were used to interpret the ICC values: an ICC value in the range of 0.81–1.00 indicated extremely high consistency; 0.61–0.80 denoted high consistency; 0.41–0.60 suggested moderate consistency; 0.21–0.40 implied poor consistency; and 0–0.20 indicated virtually no consistency (19). ICC calculations were performed using the Pingouin library in Python.

### Model construction and evaluation

Finally, using the split function in Python, the matrix data were randomly partitioned into training and test sets, with 70% of the data allocated to the training set and 30% to the test set for subsequent model construction and evaluation.

Subsequently, model construction was performed using random forests, decision tree, support vector machine (SVM), and Baidu Wenxin ERNIE models (20), respectively. During the model evaluation phase, various metrics including accuracy, precision, recall, and F1 score were calculated for each model. In addition, confusion matrices and receiver operating characteristic (ROC) curves, along with their areas under the curve, were plotted. To ensure the reliability of the model evaluation results, each model was run 10 times independently, and the run with the best performance was selected as the final model. Random forests, decision tree, and convolutional neural network models were established using Python, whereas the Baidu Wenxin ERNIE model was built on the EasyDL platform (21) (https://ai.baidu.com/easydl/).

## Results

The baseline characteristics of the 390 patients are shown in Table 2. For 50 randomly selected images, the ICC values for the radiomic feature extraction performed twice by Physician A ranged from 0.85 to 0.90. The ICC values comparing the radiomic features extracted by Physician A in the first round with those extracted by Physician B ranged from 0.80 to 0.86. These results indicated a high level of consistency in radiomic feature extraction between the two extractions performed by the same physician and between the two different physicians.

**Table 2:** Baseline characteristic data of 390 patients in this study.

**Variables**	**28F (*n* = 65)**	**32F (*n* = 65)**	**35F (*n* = 65)**	**37F (*n* = 65)**	**39F (*n* = 65)**	**41F (*n* = 65)**
Age (years)	55.71 ± 11.64	51.02 ± 13.59	50.54 ± 14.74	53.43 ± 14.20	57.86 ± 11.29	35.74 ± 14.82
Height (cm)	156.6 ± 8.23	160.78 ± 7.75	166.77 ± 9.14	169.12 ± 5.67	172.95 ± 5.44	181.86 ± 3.86
**Sex**						
Male	5(7.7%)	29(44.6%)	61(93.9%)	65(100.00%)	63(96.9%)	65(100.00%)
Female	60(92.3%)	36(55.4%)	4(6.1%)	0(0%)	2(3.1%)	0(0%)
CT model	Philips brilliance ICT 128-slice, Dual source CT					

Continuous variables are presented as mean ± SD, and categorical variables are presented as percentages.

Ultimately, Physician A completed the segmentation and radiomic feature extraction for the remaining 340 images. Subsequently, the radiomic features extracted from these 340 images were combined with those from the initial 50 images extracted by Physician A, forming a complete dataset for the final modeling.

This study employed four different algorithms to build the models and evaluate their accuracy, precision, recall, and F1 score. Table 3 presents the results of the study. By analyzing these evaluation results, it can be seen that, among the models built using the four algorithms, the Baidu Wenxin ERNIE model demonstrated the best predictive performance.

**Table 3:** Predictive performance of the four models.

	**Accuracy**	**Precision**	**Recall**	**F1-score**
Randomforests	0.62	0.62	0.62	0.61
Decision tree	0.52	0.56	0.52	0.53
Support vector machine	0.50	0.60	0.50	0.45
Baidu Wenxin ERNIE	0.77	0.77	0.78	0.77

**Figure 5:**
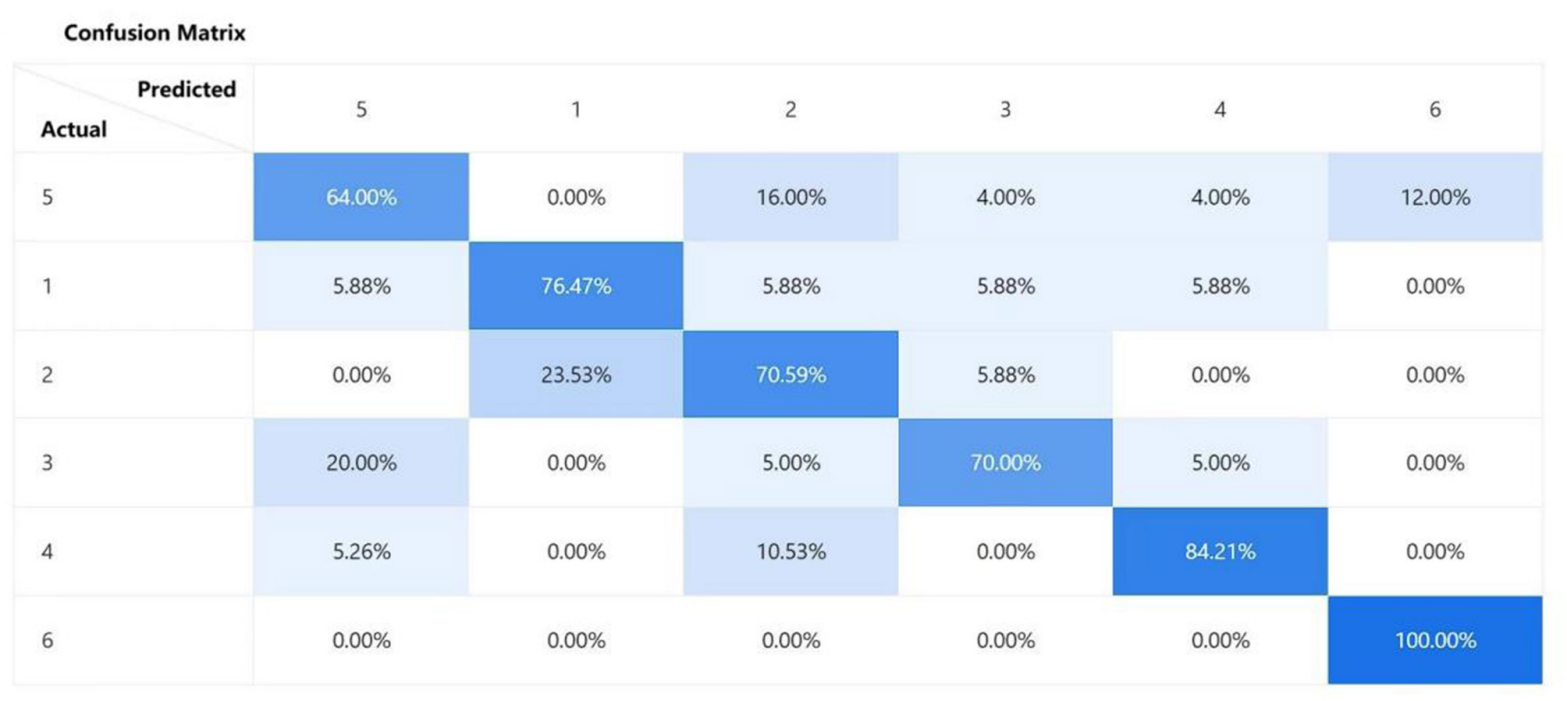
Confusion matrix plot (Baidu Wenxin ERNIE).

**Figure 6:**
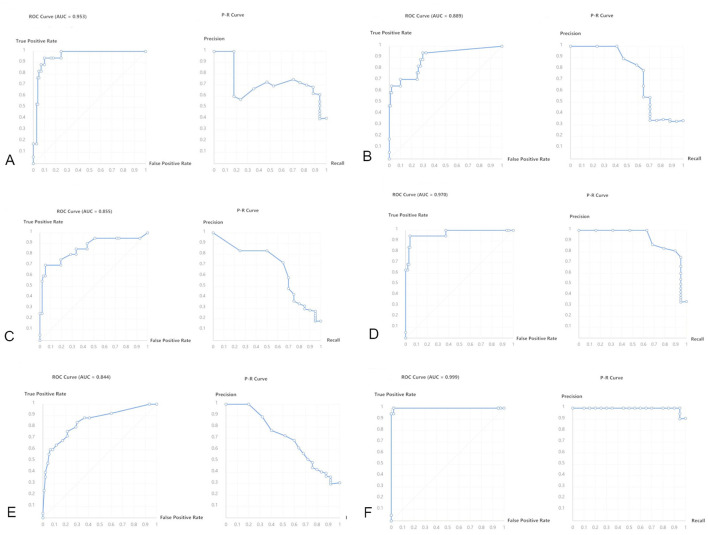
ROC and P–R curves. **(A–F)** Represent the ROC curves and Precision-Recall (P–R) curves predicted for DLT sizes 28F, 32F, 35F, 37F, 41F, respectively.

**Figure 7:**
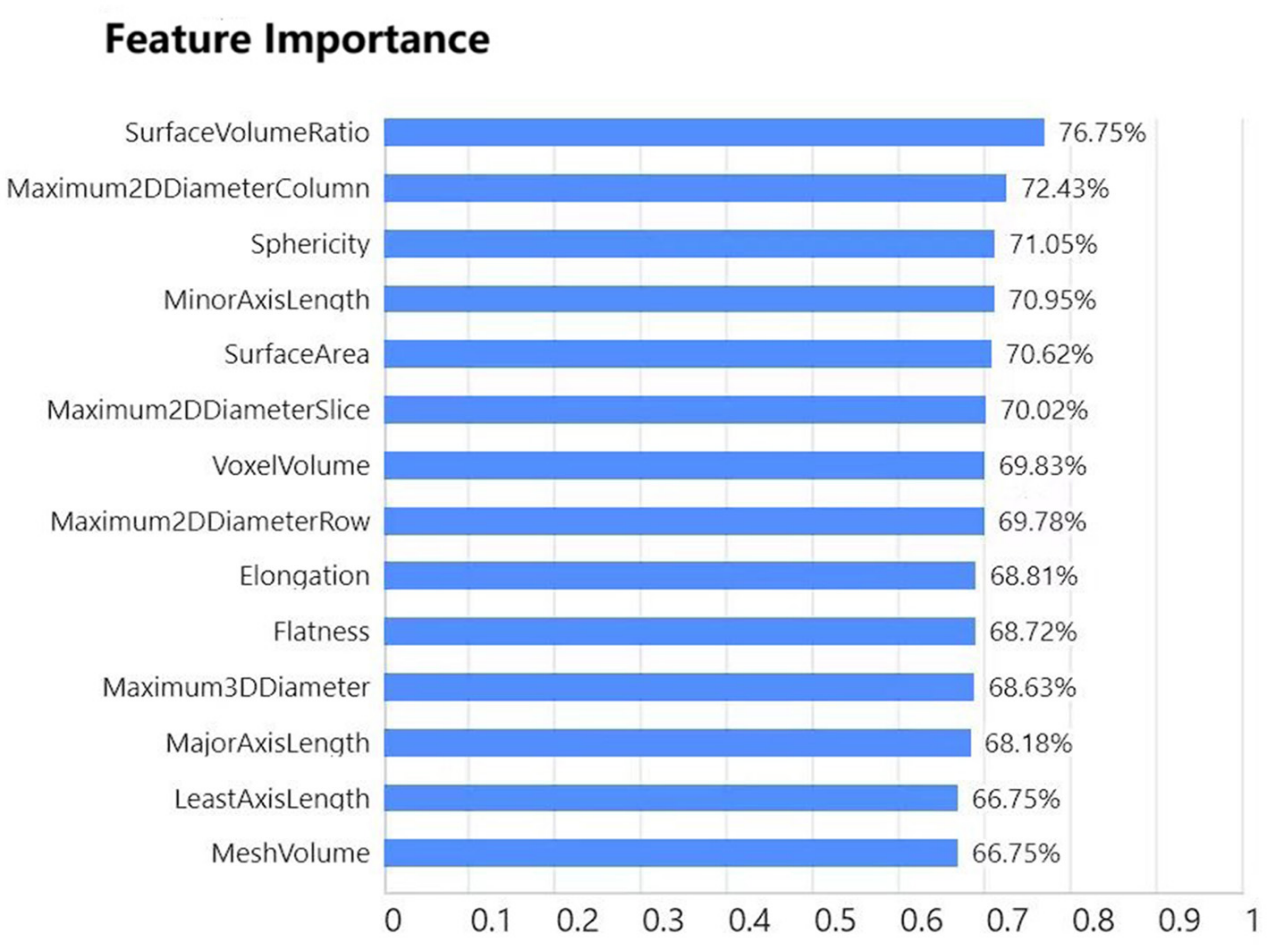
Ranking of radiomics features (Baidu Wenxin ERNIE).

To conduct a more precise and comprehensive analysis of the predictive performance of the Baidu Wenxin ERNIE model across different classification scenarios, this study employed a multidimensional analytical approach. Specifically, we meticulously constructed confusion matrices (Figure 5) to visually depict the predictive accuracy and misclassification rates of the model for each category. Additionally, we generated ROC curves (Figure 6) for each classification category to thoroughly evaluate the discriminatory ability and diagnostic efficacy of the model across various classification thresholds. Furthermore, we conducted a ranking analysis of the weights assigned to the radiomic features (Figure 7) to elucidate the roles and relative importance of each feature in the model's predictive process.

Finally, we utilized the EasyDL platform to deploy the model built based on the Baidu Wenxin ERNIE model. This initiative is aimed to facilitate other researchers by enabling them to optimize the model and conduct secondary development. The API endpoint for the model is as follows: https://aip.baidubce.com/rpc/2.0/ai_custom/v1/table_infer/4235325.

## Discussion

For anesthesiologists, the accurate selection of DLTs using CT imaging presents several significant challenges. First, there are substantial technical expertise and specialized training requirements. Anesthesiologists must possess in-depth knowledge of radiology to accurately interpret CT measurements of airway parameters such as the TD-C and the ED-C of the left bronchus. This necessitates a comprehensive understanding of CT imaging principles, image reconstruction techniques, and airway anatomy. Furthermore, proficiency in operating CT software for multiplanar reconstruction and precise measurements requires additional specialized training and practice, which may extend beyond conventional anesthesiology training curricula. Second, workflow complexity poses another hurdle. Post-CT, anesthesiologists must perform multiplanar reconstructions, meticulously adjust slice angles to achieve strict orthogonal views, and conduct precise measurements. These multistep procedures are not only time-consuming but also increase the overall workload, potentially disrupting established clinical workflows and impacting patient throughput, particularly in busy operating room environments. Therefore, the objective of this study was to develop a convenient and efficient method for radiographic assessment of DLTs using radiomics and artificial intelligence. Currently, there are two main approaches in the field of artificial intelligence-empowered medical image diagnosis. One is to directly analyze image information using deep learning algorithms, and the other is to transform medical images into radiomic features and conduct analysis and diagnosis based on these features. In this study, we adopted the second approach, which offers significant advantages by effectively circumventing the issues of high resource consumption and sluggish responses that arise when computer systems read images directly. Specifically, this method can substantially enhance the speed of image reading and analysis while significantly reducing the reliance on and consumption of graphics processing unit resources, demonstrating its immense potential and broad applicability for widespread deployment in medical settings.

In terms of algorithm selection, we opted for several widely used algorithms in the relevant field, namely, random forest, decision tree, and SVM. Among these, ensemble algorithms have demonstrated high accuracy in numerous studies related to radiomics (22–24). Given that random forest is a representative example of an ensemble algorithm, we incorporated it into our algorithm framework. One of the most prominent highlights of this study is the incorporation of the Baidu Wenxin ERNIE model. Building upon Baidu's PaddlePaddle deep learning platform and the Wenxin Knowledge-Enhanced Large Model technology, this model has demonstrated exceptional capabilities and broad application potential across multiple cutting-edge fields, including natural language processing, knowledge graph construction, and multimodal data processing. Notably, even with the relatively limited sample size in our study, the Wenxin ERNIE model enabled us to achieve the highest prediction accuracy. Furthermore, the EasyDL development platform associated with the Baidu Wenxin ERNIE model offers an intuitive and fully graphical user interface that automates the optimization of model parameters and facilitates the sharing of API interfaces. This feature significantly lowers the technical barrier, making it highly suitable for medical professionals to develop and deploy models efficiently. A considerable number of researchers in the field of Chinese medicine have utilized this platform to carry out research and achieved relatively satisfactory results (21, 25).

Based on the results depicted in the confusion matrix of the Baidu Wenxin ERNIE model, the prediction performance for the 39F was unsatisfactory, with a notable degree of error deviation. After a comprehensive analysis, we speculate that the reasons for this phenomenon are multidimensional. From a data perspective, the scarcity of the overall sample size in the training dataset may be the primary factor contributing to poor prediction outcomes. The limited sample size restricted the ability of the model to comprehensively learn the characteristic features of the 39F. Additionally, the 39F exhibited a high degree of similarity in imaging features and other aspects with the 41F. This makes it difficult for the model to distinguish between the two, thereby leading to misclassifications.

To investigate the interpretability of the Baidu Wenxin ERNIE model, we plotted a feature importance chart. Through an in-depth analysis, we found that the top three features in terms of weight were SurfaceVolumeRatio, Maximum2DDiameterColumn, and Sphericity. Notably, these three features can reflect the thickness-related characteristics of tubular objects from different perspectives, which aligns well with our manual method of judging tubular objects. Therefore, it can be inferred that the model has grasped the analytical rules for analyzing relevant features, such as the thickness of the tubular objects.

Although the Baidu Wenxin ERNIE model constructed in this study achieved an accuracy of 0.77 on the test set, there is still a gap between this level and the requirements for clinical application. Therefore, the accuracy of the model must be further improved. The trachea exhibits a degree of elasticity. In the case of small errors, the impact on clinical use may not be significant. However, if the error is large, it may lead to serious problems such as an inability to intubate smoothly or a poor sealing effect. Therefore, subsequent research should expand the sample size to enhance the generalizability and accuracy of the model. Simultaneously, more professional artificial intelligence experts are required to assist in the optimization of hyperparameters.

Despite the above-mentioned limitations, this study provides a feasible approach for the future selection of double-lumen endotracheal tubes using imaging technology. In addition, the range of ROI extraction was clearly defined, and the consistency of multiple extractions was high. This makes it suitable for combination with ROI automatic extraction software to achieve one-click extraction and result calculations. Subsequently, it can be embedded into imaging or anesthesia information systems for convenience in clinical practice.

## Conclusion

In this study, we focused on predicting tracheal tube sizes for adult double-lumen endotracheal intubation. We innovatively and precisely extracted the radiomic features of the airway from CT imaging data. Subsequently, a prediction model was constructed using the Baidu Wenxin ERNIE intelligent algorithm. The model demonstrated a favorable predictive performance, achieving an accuracy of 0.77 on the test set. The findings of this study provide a solid foundation for the application of this predictive model in clinical practice. Based on this study, it is anticipated that a comprehensive and independent modular system will be developed. This system offers clinicians a simple, rapid, and efficient method for airway assessment, thereby facilitating the efficient execution of clinical diagnosis and treatment.

## Data Availability

The raw data supporting the conclusions of this article will be made available by the authors, without undue reservation.
